# Exploring Lead Zirconate Titanate, the Potential Advancement as an Anode for Li-Ion Batteries

**DOI:** 10.1021/acsomega.4c00090

**Published:** 2024-04-17

**Authors:** Mohan K. Bhattarai, Shweta Shweta, Sunny Choudhary, Harry M. Meyer, Bishnu P. Thapaliya, Brad R. Weiner, Ram S. Katiyar, Gerardo Morell

**Affiliations:** † Department of Physics, University of Puerto Rico, San Juan, Puerto Rico 00931, United States; ‡ Department of Chemistry, University of Puerto Rico, San Juan, Puerto Rico 00931, United States; § Chemical Sciences Division, Oak Ridge National Laboratory, Oak Ridge, Tennessee 37831, United States

## Abstract

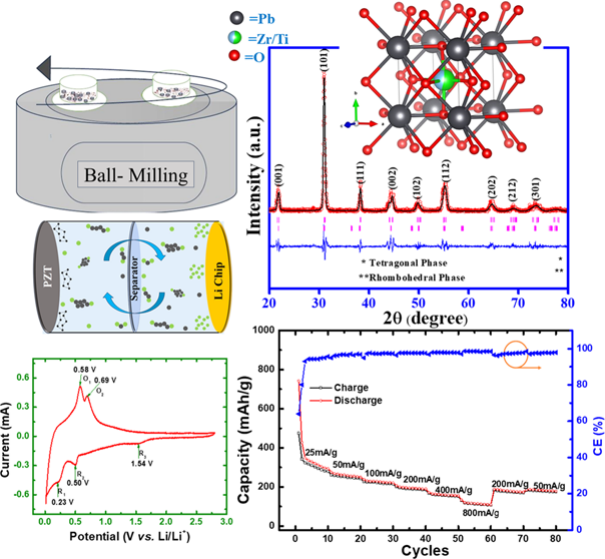

Graphite, widely adopted as an anode for lithium-ion batteries (LIBs), faces challenges such as an unsustainable supply chain and sluggish rate capabilities. This emphasizes the urgent need to explore alternative anode materials for LIBs, aiming to resolve these challenges and drive the advancement of more efficient and sustainable battery technologies. The present research investigates the potential of lead zirconate titanate (PZT: PbZr_0.53_Ti_0.47_O_3_) as an anode material for LIBs. Bulk PZT materials were synthesized by using a solid-state reaction, and the electrochemical performance as an anode was examined. A high initial discharge capacity of approximately 686 mAh/g was attained, maintaining a stable capacity of around 161 mAh/g after 200 cycles with diffusion-controlled intercalation as the primary charge storage mechanism in a PZT anode. These findings suggest that PZT exhibits a promising electrochemical performance, positioning it as a potential alternative anode material for LIBs.

## 1 Introduction

Lead zirconate titanate (PZT) with the general formula PbZr_*x*_Ti_1–*x*_O_3_ (0 ≤ *x* ≤ 1) is a perovskite-type material renowned for its intriguing ferroelectric, piezoelectric, and dielectric properties, which have been investigated for various applications, such as memories, sensors, and energy storage systems. The properties of PZT can be tailored by adjusting the ratio of Zr to Ti. When the Zr/Ti composition approaches equivalence, it is termed the morphotropic phase boundary (MPB), which holds significant importance in optimizing the dielectric, piezoelectric, and ferroelectric characteristics of these materials that exhibit their best performance.^1,2^ However, there have been limited investigations regarding the electrochemical behavior of lead-based systems as anode materials in rechargeable batteries.^3^ This research explores the possible application of PZT as an alternative anode material for lithium-ion batteries (LIBs). Graphite is a widely used and well-established anode material in LIBs. It has been the dominant choice for lithium-ion battery anodes since the commercialization of LIBs due to its excellent electrochemical properties and stability.^4,5^ However, it faces challenges such as an unsustainable supply chain and sluggish rate capabilities.^6^

Metal oxides have gained significant interest as anode materials for LIBs due to their high theoretical specific capacities, which have the potential to deliver higher energy storage capacities compared to those of traditional graphite anodes. Tin oxides (SnO_2_, SnO, Sn_2_O_3_, Sn_3_O_4_)^7,8^ tin oxide/iron composite (SnO–Sn_2_Fe),^9^ titanium oxide (TiO_2_),^10^ iron oxides (Fe_2_O_3_, Fe_3_O_4_),^11^ nickel oxide (NiO),^12^ bismuth oxide (Bi_2_O_3_),^13^ antimony oxides (Sb_2_O_3_, Sb_2_O_4_),^14^ manganese oxides (Mn_2_O_3_, Mn_3_O_4_, MnO_2_),^15^ niobium oxides (Nb_2_O_5_, NbO_2_),^16^ titanium niobium oxide (TNO),^17^ tungsten oxide (WO_3_),^18^ etc., have been explored as an anode in LIBs. In addition, perovskite oxides (ABO_3_) exhibit a range of properties, including a high dielectric constant, elevated polarization, and piezoelectricity, rendering them suitable for various applications, such as high-energy storage, memory, sensors, etc. The ABO_3_ structure, where A and B are cations, forms a three-dimensional network with O atom.^19^ Perovskite oxides, with their inherent piezoelectric behavior, offer the advantage of a versatile crystal structure that can be customized to enhance various properties, including conductivity, lithium-ion diffusivity, and structural stability.^19,20^ Ferroelectric materials could be used as electrodes in batteries. The reversible polarization of these materials accelerates lithium-ion diffusion^21^ and could lead to enhanced performance in terms of charge and discharge cycles. Some ABO_3_ materials such as strontium titanate (SrTiO_3_),^22^ barium titanate (BaTiO_3_),^23^ bismuth ferrites (BFO: BiFeO_3_),^24^ and lead titanate (PTO: PbTiO_3_)^25^ that have been already explored as an anode material for Li/Na-ion batteries. The well-known ferroelectric perovskite lead zirconate titanate (PZT) could be an alternative anode material because of its intrinsic structural properties. The versatile characteristics of PZT have attracted significant attention for their potential use in lithium-ion batteries (LIBs) as anode materials. In PZT, the reaction involves the exchange of eight Li ions and alloying with Pb:
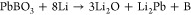
where B = B^4+^ = Zr_0.53_Ti_0.47_. This reaction yields a theoretical capacity of ∼700 mAh/g for a lithium half-cell.

Hence, this report outlines the synthesis of a high-purity PZT compound using the solid-state method and investigates its morphological structure and electrochemical performance in Li-ion insertion. The investigation revealed that PZT demonstrates a high discharge capacity of 686 mAh/g, maintaining a stable charge–discharge capacity of ∼161 mAh/g at 50 mA/g over 200 cycles (1C = 700 mA/g), accompanied by a high Coulombic efficiency (CE). The investigation points toward a diffusion-controlled intercalation mechanism as the primary charge storage mechanism, highlighting superior reversible capacity. Overall, the electrochemical performance indicates that PZT holds promise as a potential alternative anode material, offering valuable insights into the broader utilization of ferroelectric materials in LIBs.

## 2 Materials and Methods

### 2.1 Synthesis of Lead Zirconate Titanate

A conventional solid-state technique was employed to synthesize PbZr_0.53_Ti_0.47_O_3_ (PZT) powder. High-purity oxide materials, including lead oxide (PbO: Alfa Aesar; 99.9%), zirconium oxide (ZrO_2_: Alfa Aesar; 99.5%), and titanium oxide (TiO_2_: Alfa Aesar; 99.8%), were used as starting precursors. The mixture was then subjected to low-energy ball milling using zirconia balls for 24 h to thoroughly mix and homogenize the oxide powders utilizing methanol media as solvent. The mixture obtained after ball milling was subsequently dried to remove the solvent (methanol). The dried mixture was finely crushed using a mortar and pestle, ensuring that the resulting powder was well dispersed and uniform. The finely ground mixture was placed in a closed alumina crucible and calcined at 1100 °C for 10 h in a Carbolite HTF1700 furnace. This calcined powder was used for electrode fabrication.

The calcined PZT powders were crushed and well mixed with 5 wt % poly(vinyl alcohol) (PVA) as a binding agent. The PVA solution was prepared in distilled water. The mixture was then pressed into thick pellets with a diameter of 13 mm, applying a uniaxial pressure of ∼4.5 × 10^4^ Pa to achieve proper compaction and shaping. This prepared pellet was used for the ferroelectric measurement (*P*–*E* loop).

### 2.2 Anode Fabrication and Coin Cell Assembly

The mixture of PZT powder and carbon black (CB) was subjected to high-energy ball milling using zirconia balls, which is crucial in achieving the desired properties and homogeneity. The high-energy environment helps to break down the powder particles and achieve a uniform distribution of black carbon within the PZT matrix.

A 3 wt % water-soluble binder that contains a 2:1 weight ratio of sodium carboxymethyl cellulose (CMC) and styrene–butadiene rubber (SBR) was prepared in deionized water. Hereafter, we denote this binder as CMR. Then, the slurry was prepared using a mortar and pestle, ensuring even distribution of all components in a specific ratio, PZT (70%), CB (20%), and CMR (10%) solution. The prepared slurry was homogeneously spread over on a 9 μm Cu sheet using the Doctor blade machine (MTI corporation) that helped in achieving a uniform and controlled thickness of the slurry on the Cu sheet and then placed in a vacuum oven furnace for 16 h at 60 °C. The die cutter with a 10 mm diameter (MTI corporation) was used to punch the electrodes and transferred into the Ar-filled Glove box (MBraun) with water and oxygen levels <0.5 ppm for coin cell assembly. The coin cells were fabricated using a PZT electrode as a working electrode, Celgard 2400 as a separator, and a lithium chip (thickness ∼0.6 mm, MSE supplies) as a reference and counter electrode in a half-cell configuration. Cathode cap (CR2032, 18 mm), anode cap (CR2032), spring, and spacer from Landt instrument; materials SS304, a PP separator (2400 Celgard, 16 mm). 1 M lithium hexafluorophosphate (LiPF_6_; Sigma-Aldrich) in ethylene carbonate (EC)/dimethyl carbonate (DMC) (in a 1:1 volume ratio) was used as electrolyte.

### 2.3 Material Characterization

A powder X-ray diffraction (XRD) pattern was collected by employing a Rigaku Ultima III X-ray diffractometer. Rietveld refinement was used to fit the XRD pattern to determine the phase purity and structural orientation of the as-synthesized PZT powder. The X-ray source utilized Cu Kα radiation with a wavelength (λ) = 1.5405 Å configured in Bragg–Brentano (θ–2θ) geometry and operating at 40 kV and 44 mA. Scanning electron microscopy (SEM) images and energy-dispersive spectroscopy (EDS) spectra were acquired using a JEOL JEM-1400Plus manufactured by JEOL, Peabody, MA. The microscope was operated at an acceleration voltage of 120 kV (kilovolts) equipped with a LaB_6_ thermionic source. The Horiba-Jobin T64000 spectrometer was used for Raman spectroscopy. Additionally, the elemental compositions of the bulk PZT were analyzed by X-ray photoelectron spectroscopy (XPS) using a monochromated microfocusing Al Kα X-ray source of 1486.6 eV (Thermo Scientific, Model K-Alpha instrument, Waltham, MA). Raman spectra were recorded in the backscattering geometry. A confocal microscope featuring an 80× objective with a numerical aperture of 0.9 was used in conjunction with the Raman spectrometer. The small focus spot size was maintained below 3 μm, and the power of the incident laser beam for excitation was set at 2.15 mW. A Radiant tester was used to measure the electric field polarization (*P*–*E*) hysteresis.

Galvanostatic charge–discharge cycling was conducted using the multichannel battery test system CT2002A from Landt (Vestal, NY) in a voltage range of 0.01–2.8 V (vs Li/Li^+^) at different current densities. Cyclic voltammetry (CV) tests at various sweep rates (0.1–0.8 mV/s) were performed to assess redox activity, reversibility, and stability during charge–discharge cycles of the battery utilizing an Arbin instrument. Electrochemical Impedance Spectroscopy (EIS) measurements were carried out before and after cycling at the open circuit voltage (OCV). A small amplitude alternating current (AC) signal of 10 mV was employed, covering a wide range of frequencies from 0.01 Hz to 1 MHz. These measurements were performed using an interconnected setup between a Gamry potentiostat and an Arbin instrument.

## 3 Results and Discussion

### 3.1 Morphological and Ferroelectric Properties

**Figure 1:**
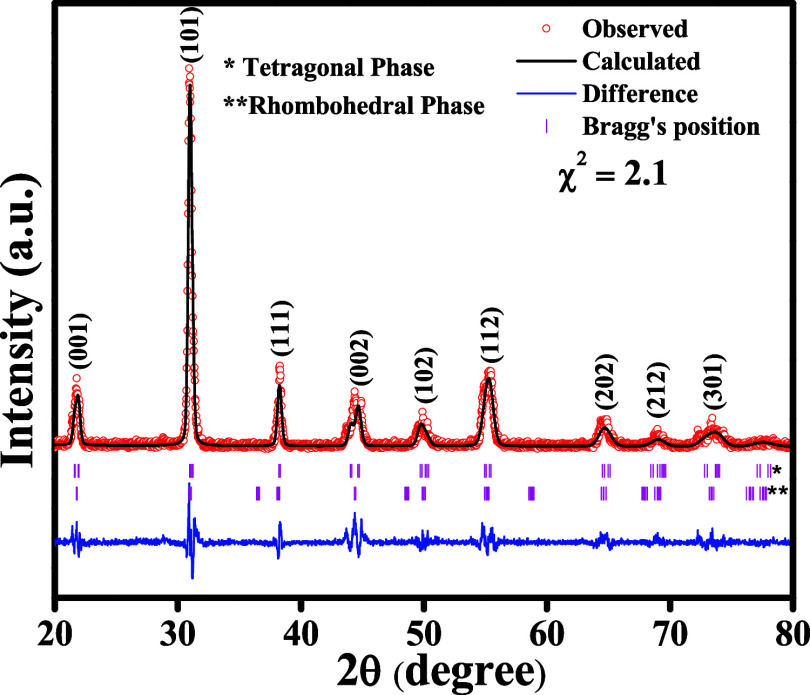
Rietveld refinement of XRD for the PZT bulk.

Figure 1 illustrates the XRD patterns of as-synthesized PZT samples, which confirmed the presence of a perovskite structure phase. The Rietveld refinement analysis revealed a predominance of the tetragonal phase, with the existence of the rhombohedral phase being less than 4%. A previous study has noted the coexistence of various phases in such a composition.^26^ Specifically, the XRD peaks corresponding to the tetragonal phase were detected at 2θ values of 21.6, 31.02, 38.3, 44.6, 49.83, 55.07, 64.5, 68.63, and 73.69°, corresponding to the (001), (101), (111), (002), (102), (112), (202), (212), and (301) crystallographic planes, respectively, as depicted in Figure 1. Additional diffraction peaks observed at 2θ values of 21.8, 31.07, 44.65, 50.4, and 64.7° corresponded to the (100), (110), (200), (201), and (220) planes, signifying the presence of the rhombohedral phase. The tetragonal phase was identified to possess a crystal structure of *P*4*mm* with lattice parameters *a* = 4.0567(08) Å and *c* = 4.1093(12) Å.

Furthermore, the application of the Rietveld refinement method unveiled evidence of a rhombohedral phase with hexagonal symmetry (*R*3*m*) characterized by lattice parameters *a* = 5.765 (3) Å and *c* = 14.182 (5) Å, in addition to the tetragonal phase. It was noted that the tetragonal phase was the predominant one. This observation aligns well with previous studies on perovskite phases and with the standard reference data for the tetragonal (JCPDS # 33-784) and rhombohedral (JCPDS # 73-2022) phases.^27−29^Table S1 provides details of the computed lattice parameters, volume (*V*), and density for the tetragonal and rhombohedral phases.

**Figure 2:**
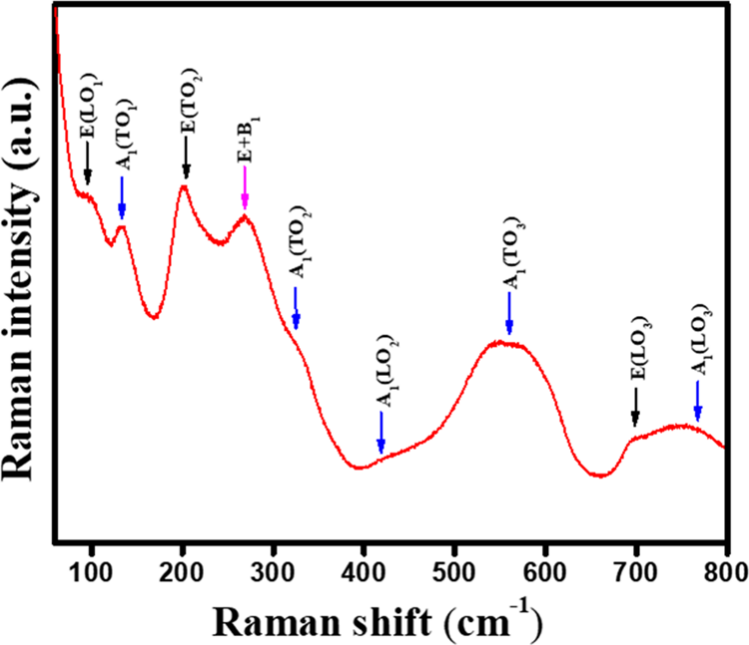
Raman spectra of PZT at room temperature.

Raman spectroscopy, recognized for its high sensitivity, investigated the lattice vibration modes. The Raman spectrum (Figure 2) showed a series of broad, overlapping bands, a characteristic feature observed in samples with a tetragonal phase within this composition range.^30^ Examining the group characteristics reveals that the tetragonal configuration of PZT possesses a collective 12 optical normal modes. Specifically, within the framework of tetragonal symmetry identified by the space group *C*_4_*_V_*^1^, these optical vibrational modes can be expressed as 3T_1u_ + T_2u_ irreducible representations. Each T_1u_ mode transforms into A_1_ + E irreducible representations, while the T_2u_ mode transforms into E + B_1_ modes. Notably, the A_1_ and E modes are both Raman active and infrared active, whereas the B_1_ mode exhibits Raman activity.^31,32^ The long-range electrostatic force eliminates the double degeneracy of a transverse mode (TO) and a longitudinal mode (LO). The low-frequency phonon modes E(LO_1_) and A_1_(TO_1_) are observed at approximately 92 and 132 cm^–1^, respectively. Additional peaks are evident at 202, 272, 324, 419, 562, 691, and 768 cm^–1^. These peaks are attributed to E(TO_2_), B_1_ + E, A_1_(TO_2_), A_1_(TO_2_), A_1_(TO_3_), E(LO_3_), and A_1_(LO_3_) modes, respectively.^33,34^ The modes E(TO_2_) and B_1_ + E are associated with BO_6_ (octahedral symmetry) rotation, while A_1_(TO_3_) and A_1_(LO_3_) are linked to O–B–O bending and B–O stretching of the oxygen octahedra, respectively.^35^

**Figure 3:**
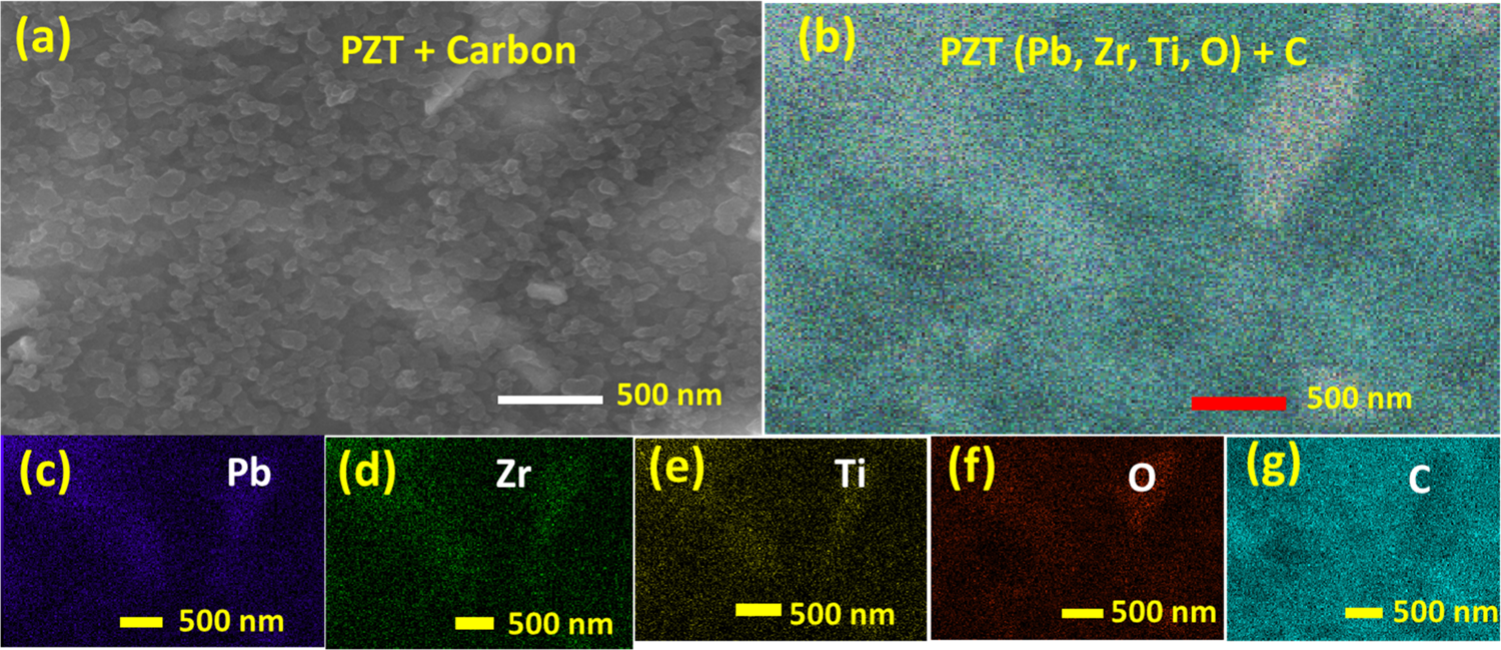
(a) SEM image and (b–g) EDX mapping of the PZT/CB composite.

Figures S1a and 3a present PZT and PZT electrode SEM images, respectively. The SEM micrograph of PZT revealed pores and well-defined granular structures consisting of randomly oriented grains (crystallites) and average grain size ∼(70–90) nm. The PZT electrode exhibited that the carbon was well distributed throughout the sample. Further elemental analysis was conducted on PZT and PZT-CB composite using EDS techniques, and the resulting spectra are displayed in Figure S3a and c, respectively. The characteristic X-ray emission lines detected in the spectrum are as follows: Pb: Mβ 5.076 keV, Pb: Lα 10.552 keV, Zr: Lα 2.042 keV, Ti: Kα 4.508 keV, O: Kα 0.525 keV for PZT, and for PZT-CB, C: Kα; 0.277 keV along with the PZT spectra. The elemental mapping [Figure 3b–g] clearly shows the homogeneous distribution of carbon with the PZT matrix. The elemental mapping of PZT is shown in Figure S1b–f. Moreover, Figure S2b presents wide-scan spectra covering the binding energy range of 0–600 eV derived from XPS analysis of PZT. The survey spectra of the two PZT surfaces reveal prominent characteristic peaks corresponding to the Pb 4f, Zr 3d, Ti 2p, and O 1s core levels.^36,37^ The O 1s peak observed at ∼531 eV is attributed to oxygen atoms bonded within the PZT material.^38^ This peak represents the energy required to excite electrons from the 1s orbital of oxygen atoms involved in chemical bonding within the PZT lattice structure. Additionally, the atomic composition (atom %) of the PZT was determined, and by eliminating the carbon atomic percentage (a surface contaminant), the elemental compositions were found to be as follows: Pb (18.5 atom %), Zr (8.6 atom %), Ti (8.5 atom %), and O (64.4 atom %).

A ferroelectric capacitor configuration of Ag/PZT/Ag was established to confirm the ferroelectricity. Electric field polarization (*P*–*E*) loop measurements affirmed the ferroelectric properties, illustrating a saturated polarization (*P*_s_) of ∼28 μC/cm^2^ under an applied electric field of ∼43 kV/cm. The accompanying figure depicted the ferroelectric loop with a remnant polarization (*P*_r_) of ∼9 μC/cm^2^ and a coercive electric field (*E*_C_) of ∼14 kV/cm. These parameters are better than those reported in the literature^26^ with a well-saturated polarization loop. The polarization observed in ferroelectric (or dielectric) capacitors and batteries corresponds to a separate phenomenon. In ferroelectric capacitors, the alignment of charges within an insulating material takes place, whereas batteries store and release energy through electrochemical reactions. Despite their distinct nature, investigating possible correlations in their polarization behaviors may offer valuable insights into enhancing energy storage technologies or optimizing the efficiency of both systems. A recent review has underscored the advanced utilization of piezoelectric materials in electrochemical processes.^20^

### 3.2 Electrochemical Performance

**Figure 4:**
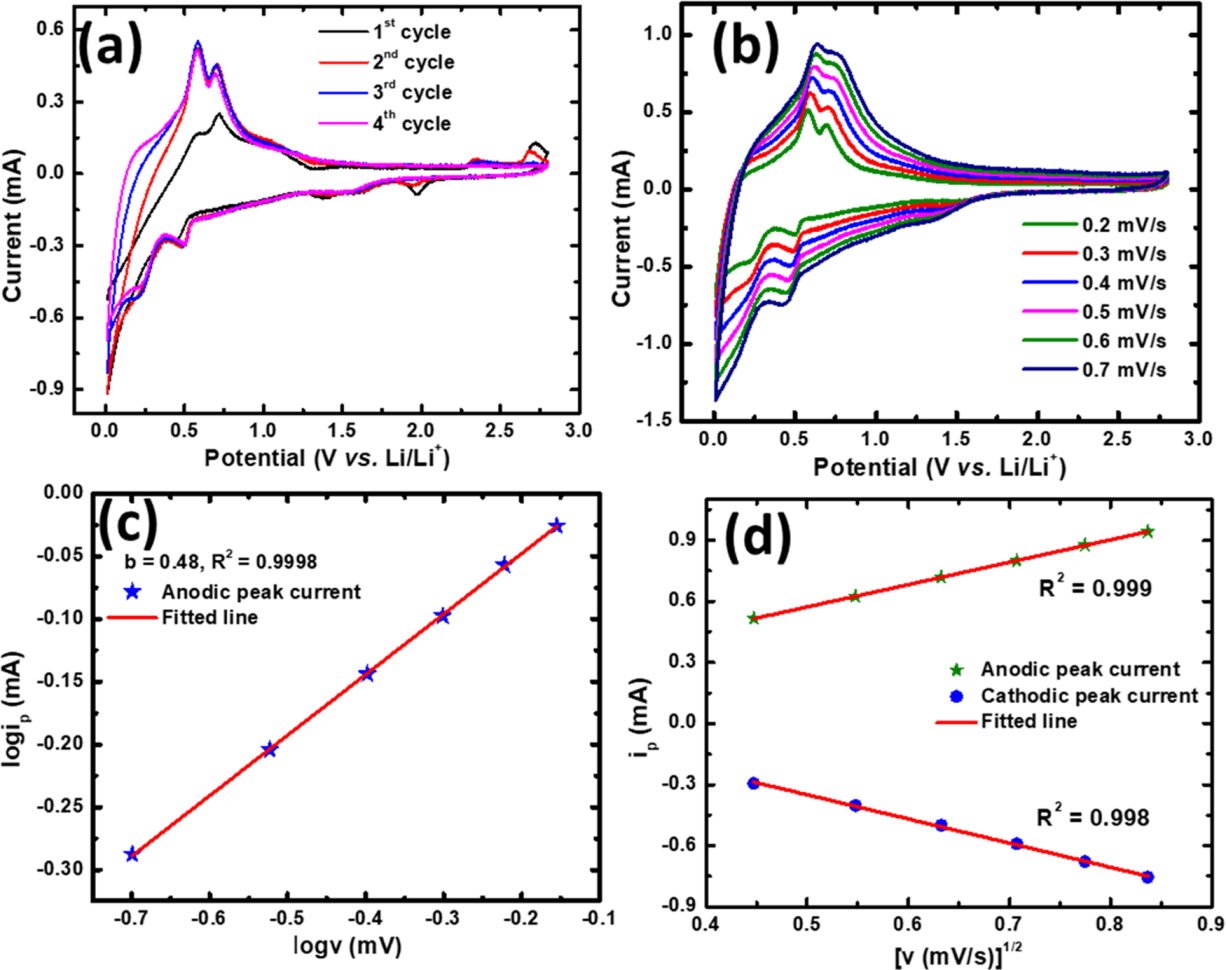
(a) CV curves for PZT at 0.2 mV/s scan rate curves (first four cycles), (b) CV curve at different scan rates from 0.2 to 0.7 mV/s, (c) log(ν) vs log(*i*_p_), and (d) scan rate (ν) vs peak current (*i*_p_).

CV analysis was conducted to understand the Li-insertion mechanism in PZT. The CV curves at 0.2 mV/s do not completely overlap in the initial cycle (Figure 4a), suggesting the formation of the solid-electrolyte interphase (SEI) and possibly structural changes in the electrode material. Following the initial activation, cyclic reversibility commenced from the second cycle onward. The presence of two distinct oxidation peaks at 0.58 and 0.69 as well as reduction peaks at 0.23 and 0.50 indicates an apparent stepwise reaction mechanism [Figures 4a and S4]. A detailed investigation is required to decipher the charge storage mechanism and electrochemical reaction pathways of the Zr-doped lead–titanium-based perovskite structure, which is beyond the scope of the present study.

Multiscan rate CVs were investigated to understand the charge storage mechanism in PZT electrodes (Figure 4b). A power-law relationship (eq 1)^39,40^ is often used to analyze the dependence of peak current (*i*_p_) on scan rate (ν) in electrochemical studies.
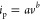
1The variable “*a*” represents a prefactor that incorporates experimental and material-specific constants, and the exponent *b* is the slope of the plot of log *i*_p_ vs log ν, a crucial parameter that provides insights into the charge storage kinetics. The value of *b* = 1 indicates a surface-controlled capacitive reaction, whereas *b* = 0.5 reflects the diffusion-controlled intercalation.^41^ In PZT, *b* = 0.48 signifies diffusion-controlled intercalation as the primary charge storage mechanism, which aligns with literature findings.^42^

The lithium-ion diffusion coefficients, *D*_Li_ (cm^2^/s), were calculated from multiscan CVs employing the Randles–Sevcik eq 2, which allows the determination of their quantitative diffusion value.^43^
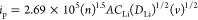
2where *i*_p_ (A) is peak current, *n* indicates the number of electrons participating in the electrode reaction (*n* = 2 for PZT), *C*_Li_^_+_^ represents the concentration of lithium ions (1.0 × 10^–3^ mol/cm^3^ for the electrolyte), *v* denotes the sweep rate of the cyclic voltammetry (V/s), and *A* corresponds to the contact area of the electrode (0.785 cm^2^). The constant 2.69 × 10^5^ (C/mol·V) is a factor in the equation.

*D*_Li_ in PZT determined using eq 2 was 3.39 × 10^–12^ cm^2^/s for deintercalation and 3.96 × 10^–12^ cm^2^/s for intercalation of lithium-ion in PZT. These similar lithium coefficients for both de/intercalation reactions suggest the highly reversible charge storage in the PZT electrode. The findings align with a result that has been reported earlier.^17^ The CV measurements, as illustrated in Figure S5, were conducted repeatedly at a constant scan rate of 0.2 mV/s after 200 cycles. The remarkable overlap observed in each cycle indicates a substantial degree of reversibility, impressive stability, and minimal degradation or change in electrode materials. The kinetics of these reactions remain uniform, underscoring the reliability of the electrochemical processes.

**Figure 5:**
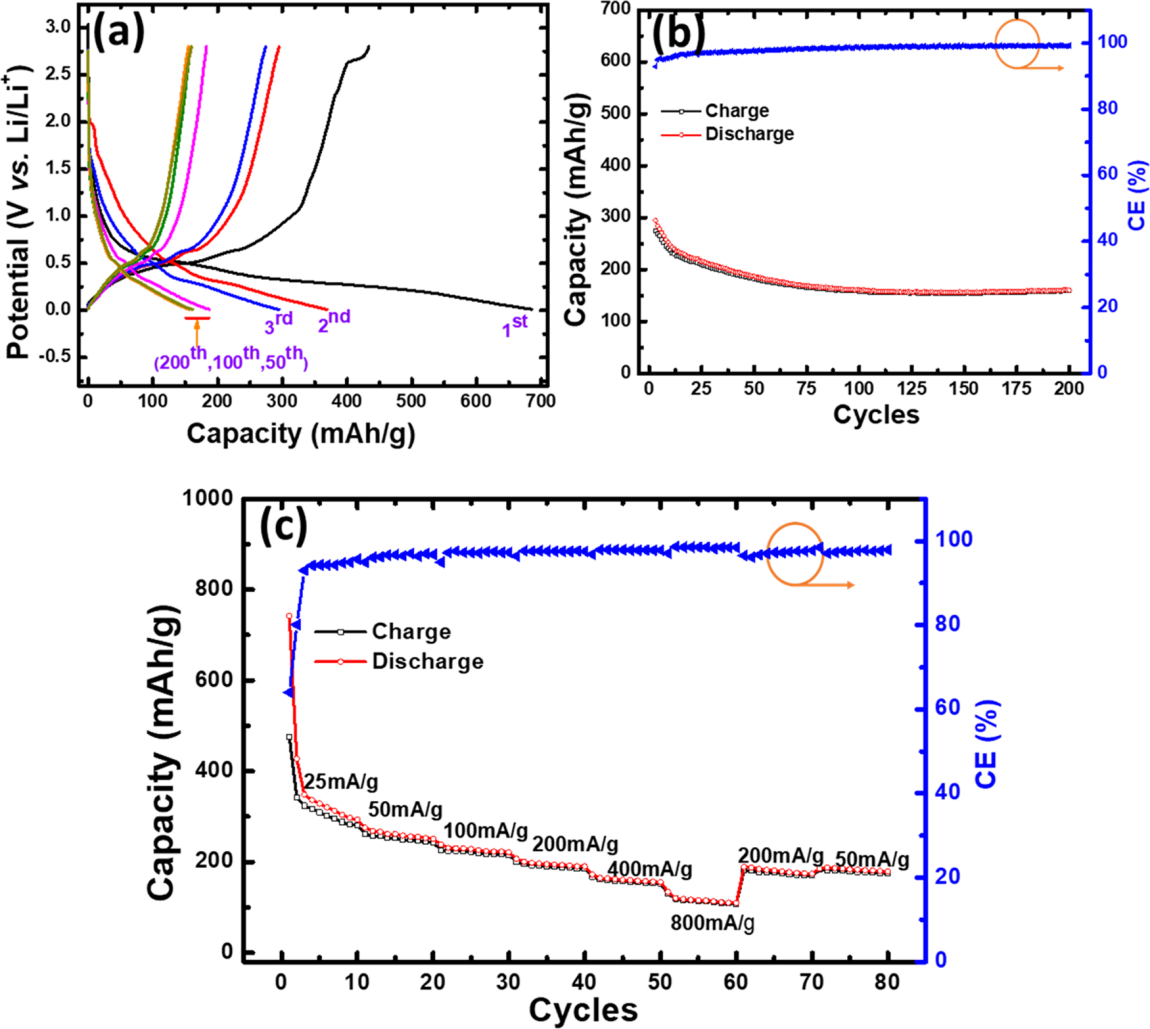
(a) Charge–discharge (vs capacity) profile of PZT at current rate 50 mAh/g for 200 cycles, (b) capacity vs cycles (3–200 cycles), and (c) rate performance.

To examine the cycling performance of PZT electrodes, galvanostatic charge/discharge cycles were conducted within a voltage range of 0.01–2.8 V, applying a constant current rate (CD) of 50 mA/g. The results are illustrated in Figure 5a,b. PZT exhibited an initial discharge capacity of ∼686 mAh/g and a Coulombic efficiency (CE = charge capacity/discharge capacity) of around 63%. The significant irreversible capacity loss experienced during the initial cycle is attributed to interfacial parasitic side reactions and irreversible conversion reactions, resulting in the formation of a solid-electrolyte interface (SEI) layer. The extent of irreversible capacity loss varies based on factors such as the negative-to-positive capacity ratio, the surface area of active particles, and operational conditions.^44^ After the fourth cycle, the CE reached above 95%. The PZT anode delivered a stable 161 mAh/g capacity over 200 cycles, with an average CE of ∼99%. This outcome surpasses the reported capacity on PTO, which demonstrated a capacity of 84.2 mAh/g at a rate of 30 mA/g^25^ and comparable to the capacity of the BFO electrode reported for 100 cycles.^24^ Furthermore, the rate capability of the PZT electrode was assessed over a range of current rates, ranging from 25 to 800 mA/g as depicted in Figure 5c. Even under the high current of 800 mA/g, the battery demonstrated a capacity of approximately 112 mAh/g, suggesting that the PZT anode could be a suitable alternative for a fast-charging anode. When the current rates reverse back (200 and 50 mA/g), the cell regains its initial capacity, underscoring the cyclic resilience and the structural robustness of the PZT.

**Figure 6:**
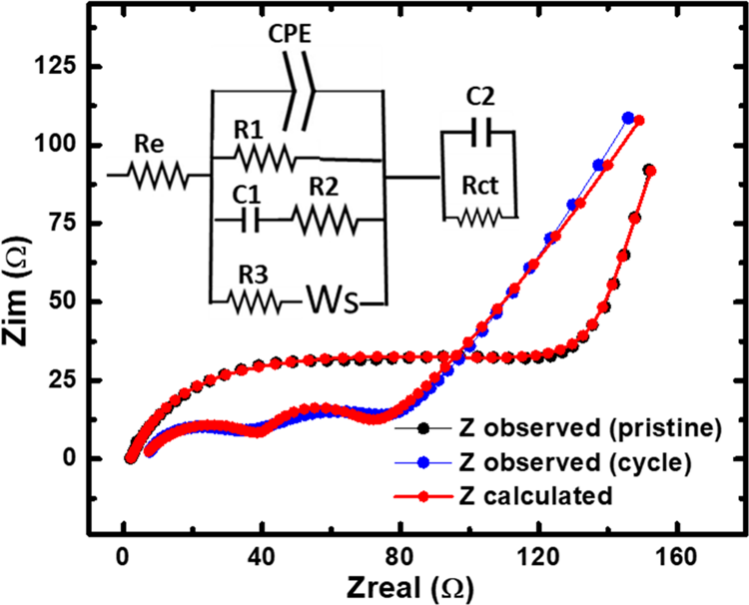
Nyquist plots of the PZT electrode before and after cycles.

Figure 6 shows the Nyquist plot of electrochemical impedance spectra of Li-PZT half-cell collected before and after cycling. The recorded EIS spectra were fitted, employing Z-SimpWin3.6 software to analyze the charge transfer characteristic parameters. In the equivalent circuit, *R*_e_ and *R*_ct_ represent the Ohmic and charge transfer resistance, respectively, corresponding to the electrolyte and the electrode.^45^ These parameters play a crucial role in investigating electrochemical reactions, which optimize battery performance, improve charging/discharging rates, and enhance the energy storage capacity of batteries and supercapacitors to ensure optimal battery operation and longevity. The slight increase in *R*_e_ and remarkably increased value in *R*_ct_ were observed before and after cycling. The observed slight increase in *R*_e_ and a significant increase in *R*_ct_ before and after cycling suggest changes in the system’s electrochemical behavior, potentially influenced by cycling-induced alterations in the electrode–electrolyte interface. Cycling-induced reactions can lead to the formation of passivation layers or surface films on the electrode surface. While these layers serve to protect the electrode from further degradation, they can hinder charge transfer, resulting in an increase in both *R*_ct_ and *R*_e_.^46^ Additionally, cycling-induced changes in the surface chemistry of the electrode, such as the adsorption of reaction intermediates or the formation of surface oxides, can influence the kinetics of charge transfer reactions.

Before cycling, the presence of a single semicircle in the Nyquist plot indicates the electrode’s straightforward electrochemical performance. However, following charge/discharge cycling, the appearance of a double semicircle suggests potential alterations in the anode materials. This change could be attributed to the increased resistance, particularly *R*_ct_, which may explain the formation of the second semicircle in the high-frequency region.^44^ Warburg element (*W*_s_) is linked to ion diffusion within the electrolyte and is represented by a sloping line on the Nyquist plot, particularly at lower frequencies. The angle and slope of the Warburg element offer valuable insights into the diffusion characteristics of the system being studied. The constant phase element (CPE) also indicates the diffusion capacitance resulting from the reactive ion diffusion process. This introduction of CPE is essential because the interface does not behave like an ideal capacitor.^47^ The impedance of the CPE, represented as *Z*_CPE_, is expressed as the following equation:^48^
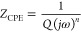
where *Q* represents the CPE constant associated with the electrode/electrolyte interface, *j* denotes the imaginary unit (√−1), ω (=2π*f*) indicates the angular frequency, and *n* is the dimensionless constant exponent of the CPE. When *n* is −1, the CPE exhibits inductive behavior; *n* = 1, the CPE acts as a pure capacitor, and equivalent to Warburg impedance (*Z*_*W*_s__) when *n* = 0.5.^49^ Further, a low CPE value may suggest that the electrodes and electrolyte in the LIBs are relatively simple and well-behaved electrode–electrolyte interfaces. The fitted parameters obtained from the RC model are summarized in Table 1.

**Table 1:** Calculated Parameters of PZT Electrode from EIS Measurement

	parameters					
condition	*R*_e_ (Ω)	*W*_s_ (Ω/s^0.5^)	*R*_ct_ (Ω)	CPE (Ω/s^*n*^)	*n*	χ^2^ (10^–4^)
before cycling	1.97	4.1 × 10^–4^	13.6	2.87 × 10^–5^	0.75	0.64
after 200 cycles	5.55	6.9 × 10^–3^	234.5	1.43 × 10^–5^	0.64	7.89

## 4 Conclusions

In this study, PZT material was successfully synthesized via the solid-state reaction method, which confirmed the phase purity by Rietveld analysis of X-ray data, showing a predominant tetragonal perovskite phase validated by Raman analysis. The electrochemical behavior of the PZT electrode was examined with cyclic voltammetry (CV), revealing a controlled ion diffusion mechanism (*b* ∼ 0.48). Additionally, the high initial discharge capacity (∼686 mAh/g) and stable capacity of ∼161 mAh/g after 200 cycles highlight its potential for delivering both high discharge capacity and stability. Furthermore, it demonstrated an increased current capability (800 mA/g). Thus, cyclic reversibility, high-rate capabilities, and stable capacity emphasize the potential of PZT as an alternative anode for LIBs.

## Supporting Information Available

Table S1 presents the calculated parameters derived from the Rietveld refinement of PZT; in Figure S1, (a) shows an SEM image, while (b) depicts the EDX spectra, and (b–f) illustrates the EDX mapping of PZT; Figure S2a,c displays the EDX spectra for (a) PZT and (b) the PZT/CB, respectively, and Figure S2b displays XPS spectra of PZT; additionally, Figure S3 exhibits the ferroelectric loop of PZT; in addition, Figures S4 and S5 showcase the CV curve recorded at a scan rate of 0.2 mV/s for the PZT electrode both before cycling and after 200 cycles (PDF).
