# Supplementary material for: Exploring Lead Zirconate Titanate, the Potential Advancement as an Anode for Li-Ion Batteries
Source: ACS Omega. 2024 Apr 17;9(17):19219–26. doi: 10.1021/acsomega.4c00090 (PMC11064192; doi:10.1021/acsomega.4c00090)
Supplement: Supplementary file 1 — ao4c00090_si_001.pdf [file ao4c00090_si_001.pdf]

Supplementary Information

Exploring Lead Zirconate Titanate, the Potential Advancement as an Anode for Li-Ion Batteries.

Mohan K Bhattarai[1*], Shweta Shweta[1], Sunny Choudhary[1], Harry M Meyer III[3], Bishnu P Thapaliya[3], Brad R Weiner[2], Ram S Katiyar[1], and Gerardo Morell[1*]
[1]Department of Physics, University of Puerto Rico, San Juan, PR-00931, USA
[2]Department of Chemistry, University of Puerto Rico, San Juan, PR-00931, USA
[3]Chemical Sciences Division, Oak Ridge National Laboratory, Oak Ridge, TN, 37831, USA

*Correspondence author's email: mohankbhattarai@gmail.com and gerardo.morell@upr.edu

Table S1: Calculated parameters from Rietveld refinement of PZT

Parameters	Tetragonal Phase	Rhombohedral Phase
Space Group	P4mm	R3m
Lattice parameters (A°)	a=b= 4.0567 (08)<br>c= 4.1093 (12)	a =b= 5.765 (3)<br>c = 14.182 (5)
Volume (V) $(A°)^3$	67.628	408.136
Density (g/ cm^3)	7.61	3.07

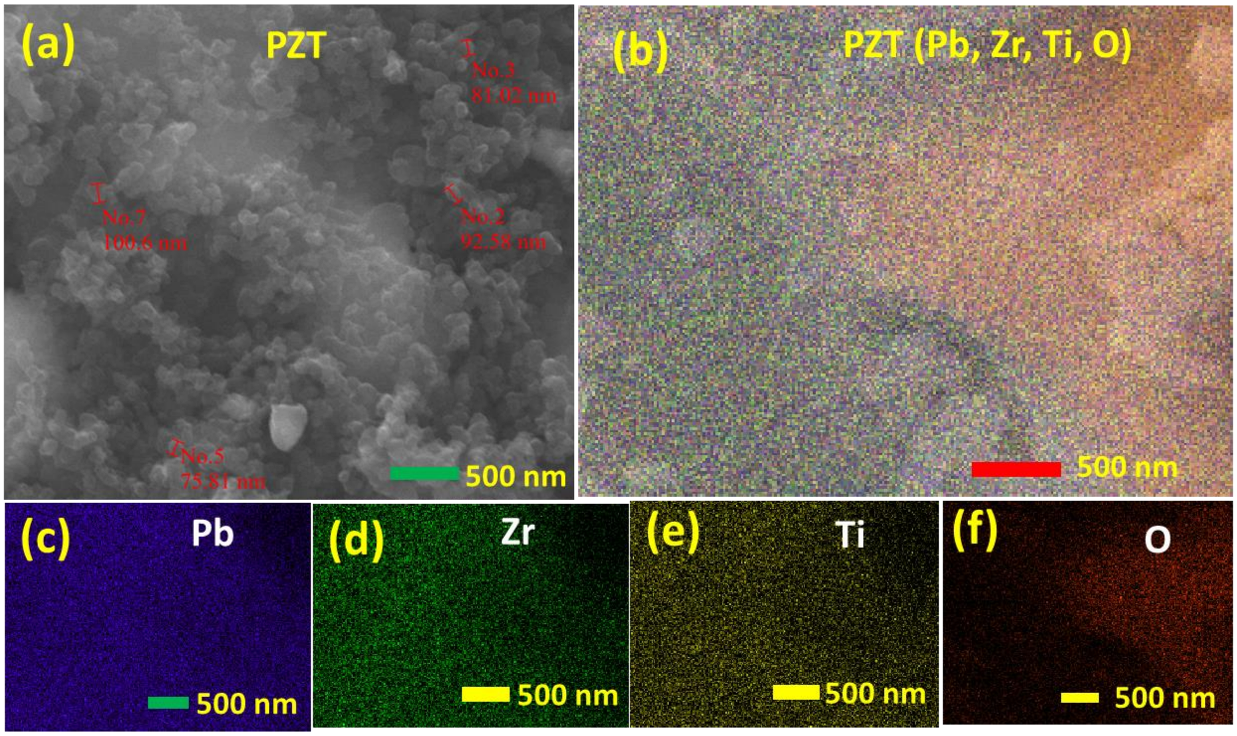

Figure: S1 (a) SEM image (b) EDX spectra (c-f) EDX mapping of PZT.

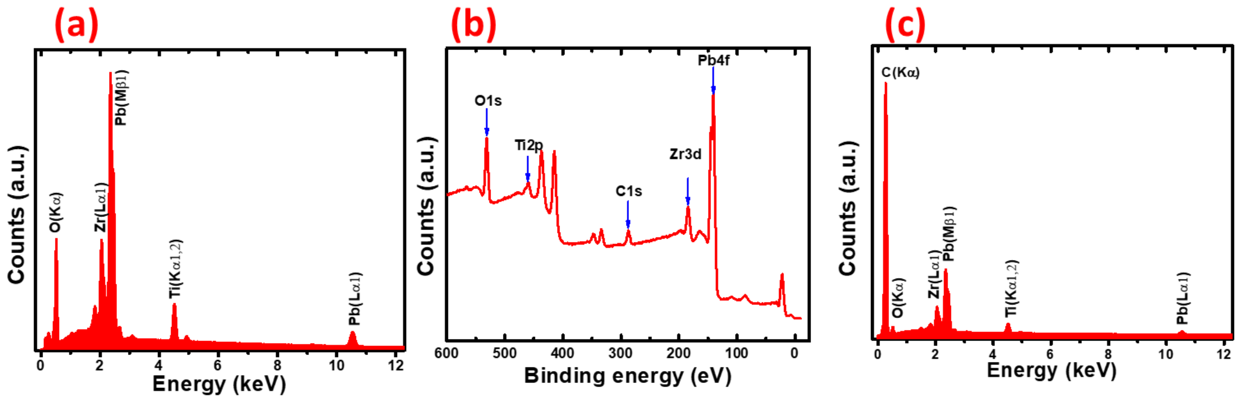

Figure: S2 EDX spectra (a) PZT (XPS spectra of PZT (c) EDS spectra of PZT/CB composite.

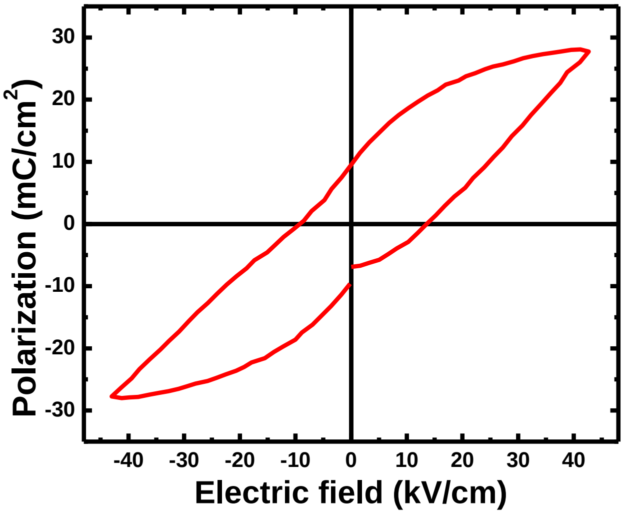

Figure: S3 Ferroelectric loop of PZT

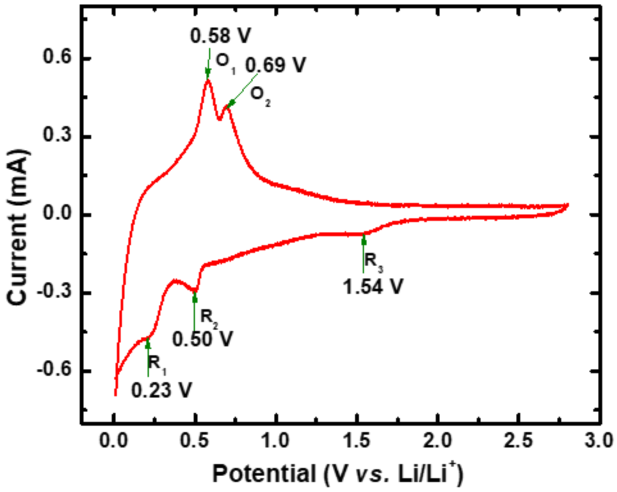

Figure: S4 CV curve recorded at scan rate 0.2 mV/s for PZT electrode before cycling.

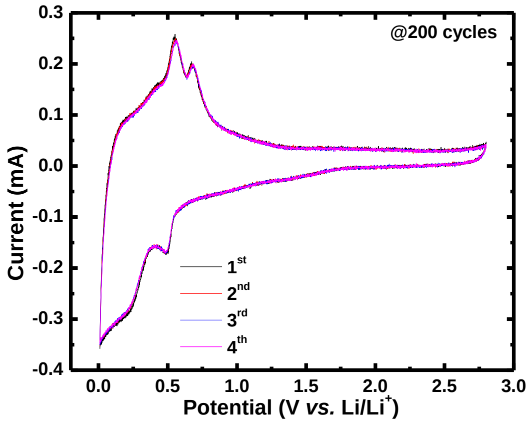

Figure: S5 CV curve recorded at scan rate 0.2 mV/s after 200 cycles for PZT electrode.
